# Optimizing Vancomycin Dosing in Chronic Kidney Disease by Deriving and Implementing a Web-Based Tool Using a Population Pharmacokinetics Analysis

**DOI:** 10.3389/fphar.2019.00641

**Published:** 2019-06-11

**Authors:** Sreemanee Raaj Dorajoo, Chrystal Leandra Winata, Jessica Hui Fen Goh, Say Tat Ooi, Jyoti Somani, Lee Ying Yeoh, Siok Ying Lee, Chun Wei Yap, Alexandre Chan, Jung-woo Chae

**Affiliations:** ^1^Department of Pharmacy, National University of Singapore, Singapore, Singapore; ^2^Department of Pharmacy, Khoo Teck Puat Hospital, Singapore, Singapore; ^3^Department of Medicine (Infectious Diseases), Khoo Teck Puat Hospital, Singapore, Singapore; ^4^Department of Medicine (Renal Medicine), Khoo Teck Puat Hospital, Singapore, Singapore; ^5^Health Services & Outcomes Research, National Healthcare Group, Singapore, Singapore; ^6^College of Pharmacy, Chungnam National University, Daejeon, South Korea

**Keywords:** population pharmacokinetics, vancomycin, chronic renal failure, prediction modelling, precision medicine

## Abstract

**Background:** Chronic kidney disease (CKD) patients requiring intravenous vancomycin bear considerable risks of adverse outcomes both from the infection and vancomycin therapy itself, necessitating especially precise dosing to avoid sub- and supratherapeutic vancomycin exposure.

**Methods:** In this retrospective study, we performed a population pharmacokinetic analysis to construct a vancomycin dose prediction model for CKD patients who do not require renal replacement therapy. The model was externally validated on an independent cohort of patients to assess its prediction accuracy. The pharmacokinetic parameter estimates and the equations were productized into a Web application (VancApp) subsequently implemented in routine care. The association between VancApp-based dosing and time-to-target concentration attainment, 30-day mortality, and nephrotoxicity were assessed postimplementation.

**Results:** The model constructed from an initial cohort (n = 80) revealed a population clearance and volume of distribution of 1.30 L/h and 1.23 L/kg, respectively. External model validation (n = 112) demonstrated a mean absolute prediction error of 1.25 mg/L. Following 4 months of clinical implementation of VancApp as an optional alternative to usual care [VancApp (n = 22) vs. usual care (n = 21)], patients who had received VancApp-based dosing took a shorter time to reach target concentrations (median: 66 vs. 102 h, p = 0.187) and had fewer 30-day mortalities (14% vs. 24%, p = 0.457) compared to usual care. While statistical significance was not achieved, the clinical significance of these findings appear promising.

**Conclusion:** Clinical implementation of a population pharmacokinetic model for vancomycin in CKD can potentially improve dosing precision in CKD and could serve as a practical means to improve vital clinical outcomes.

## Introduction

Treatment success with intravenous vancomycin hinges on timely and accurate attainment of target drug exposure (Hidayat et al., 2006; Martinez et al., 2012). Despite decades of clinical experience, accurate dosing of vancomycin remains challenging in situations of altered pharmacokinetics. Chronic kidney disease (CKD) alters both the volume of distribution (Vd) and clearance of vancomycin, predisposing patients to dosing inaccuracies which lead to delayed target exposures (Jeffres et al., 2007; Matzke et al., 2011). Inaccurate dosing is particularly hazardous in CKD patients with serious methicillin-resistant *Staphylococcus aureus* (MRSA) infections because CKD is independently associated with an elevated mortality risk (Pastagia et al., 2012). While aggressive treatment may thus be warranted in such scenarios, vancomycin-associated nephrotoxicity is also more likely to occur in patients with preexisting renal impairment (Panwar et al., 2013). Therefore, precise dosing plays a pivotal role in maximizing treatment success in CKD patients requiring intravenous vancomycin.

However, there remains a lack of models specifically designed for dosing vancomycin in CKD (Moellering et al., 1981; Matzke et al., 1984; Rodvold et al., 1988; Rybak et al., 2009; Thomson et al., 2009; Cardile et al., 2015). Although the area under the curve to minimum inhibitory concentration ratio (AUIC) is gaining popularity as the preferred parameter of efficacy, models that optimize troughs may be still useful in settings where the minimum inhibitory concentrations (MICs) are ≤1 mg/L. Typically, trough concentrations of 15 mg/L are able to achieve AUICs that exceed the target threshold of 400 mg h/L in these scenarios (Rybak et al., 2009; Pai et al., 2014). In this study, we propose a model for optimizing vancomycin therapy with a retrospective study based on trough concentrations in CKD. Additionally, we retrospectively evaluate the clinical impact of model-based dosing on clinical outcomes following implementation in routine care (Dorajoo and Chan, 2017).

## Materials and Methods

### Study Overview, Design, and Setting

This study was carried out in three phases. The first phase involved model construction and internal validation, the second phase involved external model validation and Web application development, and the third phase involved model implementation and postimplementation evaluation. The results of each phase motivated subsequent phases of the study. All study phases were approved by the Domain Specific Review Board, with a waiver of informed consent. All phases of the study were conducted at Khoo Teck Puat Hospital (KTPH), Singapore—a 600-bed tertiary healthcare institution providing a wide range of medical and surgical specialty services.

### Phase 1: Model Derivation and Internal Validation

Model derivation was performed by extracting records of patients who had received intravenous vancomycin from 1^st^ April 2013 to 31^st^ March 2014. An in-house vancomycin protocol is routinely used to dose intravenous vancomycin. Briefly, weight-based doses of 15–20 mg/kg are administered at intervals of 12 or 24 h and infused at a maximum rate of 500 mg/h. A maximum dose of 2 g is administered per infusion, regardless of total body weight. The vast majority of MRSA isolates exhibit a vancomycin MIC of 1 mg/L at KTPH.

#### Inclusion/Exclusion Criteria and Data Collection

Patients were included if they had received at least two doses of intravenous vancomycin over a 72-h period, had a baseline creatinine clearance (CrCl) of less than 60 ml/min based on the Cockcroft–Gault equation using total body weight (to mirror actual clinical practice), and at least one measured vancomycin concentration. Patients were excluded if they were receiving renal replacement therapy, had no documented weight or serum creatinine measured at baseline, or if they had previously received intravenous vancomycin less than 2 weeks before the date of therapy initiation. Details of the dosing regimen administered for the first 5 days of therapy were collected. The list of potential covariates which could influence the clearance and Vd of vancomycin is provided as **Supplementary Material** (Jelliffe, 1973; Levey et al., 1999; Levey et al., 2009).

#### Model Derivation

A population pharmacokinetic (PopPk) analysis was performed in NONMEM version 7.3. A first-order conditional estimation method with interactions (FOCE-I) was employed for the model building process (Wahlby et al., 2001; Wahlby et al., 2002). The interindividual variability (IIV) in the model was determined by exponential random effects, while the residual variability was modelled as a mixture of additive and proportional error structures. Stepwise univariate forward selection followed by backward elimination was used in covariate selection. Details of the covariate selection procedure are provided as **Supplementary Material**.

#### Internal Validation

Internal model validity was assessed *via* visual inspection of the scatter plots of plasma concentrations versus population prediction (PRED) and individual predicted (IPRED) concentrations. The relative prediction errors were graphically described by conditional weighted residuals (CWRES) and plotted against PRED and time after dose. One thousand bootstrapped datasets were generated *via* repeated random sampling with replacement to evaluate the stability of the estimates of the final model (Ette, 1997).

### Phase 2: External Validation Web Application Development

External validation was performed on a separate group of CKD patients who had received intravenous vancomycin from 1^st^ April 2014 to 31^st^ March 2015. The model was used to predict the observed vancomycin concentrations in the external cohort. Mean absolute error (MAE), mean squared error (MSE), and root mean square error (RMSE) were used to evaluate model accuracy.

(1) $$\text{MAE}=\frac{1}{n}\sum_{1}^{n}\left|\left(\text{Obs}-\text{Pred}\right)\right|$$

(2) $$\text{MSE}=\frac{1}{n}\sum_{1}^{n}{\left(\text{Obs}-\text{Pred}\right)}^{2}$$

(3) $$\text{RMSE}=\sqrt{\text{MSE}}$$

The prediction error (PE%) was used to calculate the mean absolute prediction error (MAPE%) of the model:

(4) $$\text{PE}\%=\frac{\text{Obs}-\text{Pred}}{\text{Obs}}\times 100\%$$

(5) $$\text{MAPE}\%=\frac{1}{n}\sum_{1}^{n}\left(\left|\text{PE}\%\right|\right)$$

A model with an MAPE of less than 30% was arbitrarily considered to be clinically acceptable (Zhao et al., 2016). Additionally, differences between observed and predicted concentrations were visually assessed using the Bland–Altman plot.

The model was subsequently productized into two Web applications (VancApp) to facilitate point-of-care decision-making, for initial dosing and subsequent dose adjustments. VancApp was designed to allow clinicians the flexibility of selecting appropriate loading and maintenance dose pairs for a given patient and to visualize the simulated concentration–time profile (Wojciechowski et al., 2015).

### Phase 3: Clinical Implementation and Impact Assessment

#### Clinical Implementation and Usage Protocol

VancApp fulfilled the criteria for quality improvement projects/clinical services specified by the Domain Specific Review Board and was approved for use by the hospital Medical Board in August 2016. VancApp was implemented in routine care in September 2016. Usage of VancApp followed the prevailing safety guidances as per the in-house dosing protocol (i.e., a maximum of 30 mg/kg for loading and 20 mg/kg for maintenance doses, administered at a maximum rate of 500 mg/h). VancApp’s implementation was made known to all medical and surgical teams *via* weekly multidisciplinary departmental meetings.

VancApp was integrated into the clinical workflow by engaging ward pharmacists who screened and evaluated VancApp usage eligibility. VancApp was only used in patients with a CrCl less than 60 ml/min. All pharmacists had undergone two training sessions on the appropriate use of VancApp in August 2016. Briefly, pharmacists were trained to enter the required parameters (age, gender, serum creatinine, and total body weight) to determine the most appropriate loading (if applicable) and maintenance dose pair. VancApp-based dose recommendations were then communicated to the primary team who could choose to defer from the recommendation. Pharmacists electronically documented VancApp usage to facilitate monitoring and automated data retrieval.

#### Usual Care Dosing

In the usual care group, patients were dosed according to the in-house dosing protocol at KTPH where clinical teams had chosen not to accept the VancApp dose. Typically patients with mild to moderate skin/soft tissue infections (SSTIs) as well as urinary tract infections are dosed at 10 to 15 mg/kg. Doses of 15 to 20 mg/kg are used to treat pneumonia, sepsis, bacteremia, osteomyelitis, meningitis, and severe SSTIs as per the in-house protocol (Rybak et al., 2009).

#### Clinical Impact Assessment

To assess the impact of VancApp-based dosing, we retrospectively reviewed patients who received vancomycin from November 2016 to February 2017. Baseline demographics, laboratory test data, comorbidities, indication of vancomycin, and concomitant nephrotoxin exposure were assessed. The primary outcome was the time to reach the first therapeutic target trough. Secondary outcomes were the length of hospitalization, 30-day mortality, 30-day readmission due to MRSA infections, and vancomycin-associated nephrotoxicity (defined as a 50% or greater increase in serum creatinine).

The time to attain the first target trough was compared using the Kaplan–Meier estimator and the log rank test was used to assess for differences. Differences in secondary outcomes were tested using the Mann–Whitney *U* and Fisher’s exact test where appropriate.

## Results

### Phase 1: Model Derivation and Internal Validation

The derivation cohort comprised of 80 patients who had a mean ± standard deviation (SD) CrCl of 33.8 ± 10.3 ml/min and total body weight (TBW) of 57.8 ± 15.7 kg. Seventy-five percent of patients were at CKD stage 3 and beyond. Other demographic and clinical parameters of patients are summarized by cohort in **Table 1**. In total, 170 vancomycin concentrations were acquired over the initial 120 h of therapy, amounting to an average of 2.1 ± 1.3 (range: 1–4) concentrations per patient. From the time of dose administration, there was at least one sample acquired every 2 h from one or more patients, up to the 50^th^ hour following dose administration.

**Table 1:** Baseline characteristics of subjects in the model construction and validation cohorts.

Variable	Construction (*n* = 80) 1^st^ April 2013 – 31^st^ March 2014	Validation (*n* = 112) 1^st^ April 2014 – 31^st^ March 2015
Sex, n (%)		
Male	51 (63.7)	**71 (63.4)**
Female	29 (36.3)	**41 (36.6)**
Age, years, mean ± SD	71.7 ± 13.0	**73.5 ± 12.5**
Total body weight (kg), mean ± SD	57.8 ± 15.7	**58.7 ± 14.1**
Height (m), mean ± SD	1.59 ± 0.08	**1.60 ± 0.10**
Body mass index (kg/m^2^), mean ± SD	22.6 ± 5.9	**22.7 ± 4.7**
Body surface area (m^2^), mean ± SD	1.58 ± 0.23	**1.61 ± 0.22**
Serum creatinine (µmol/L), mean ± SD	150.6 ± 73.6	**151.1 ± 83.1**
Creatinine clearance* (ml/min/1.73 m^2^), mean ± SD	33.8 ± 10.3	**35.5 ± 13.6**
Estimated glomerular filtration rate^ (ml/min/1.73 m^2^), mean ± SD	46.2 ± 23.0	**48.9 ± 28.8**
Chronic kidney disease stage^, n (%)		
CKD 1 (≥90 ml/min/1.73 m^2^)	5 (6.3)	**14 (12.5)**
CKD 2 (60–89 ml/min/1.73 m^2^)	15 (18.8)	**14 (12.5)**
CKD 3a (45–59 ml/min/1.73 m^2^)	18 (22.5)	**23 (20.5)**
CKD 3b (30–44 ml/min/1.73 m^2^)	20 (25.0)	**29 (25.9)**
CKD 4 (15–29 ml/min/1.73 m^2^)	19 (23.8)	**26 (23.2)**
CKD 5 (<15 ml/min/1.73 m^2^)	3 (3.8)	**6 (5.3)**
Serum albumin (g/L), mean ± SD	27.3 ± 6.7	**27.1 ± 6.0**
C-reactive protein (mg/L), mean ± SD	102.0 ± 81.9	**122.4 ± 95.9**
Procalcitonin (ng/ml), mean ± SD	10.1 ± 16.8	**26.5 ± 16.0**
Total white blood cell count, mean ± SD	13.76 ± 8.09	**13.87 ± 6.36**
Neutrophil percentage (%), mean ± SD	83.9 ± 9.9	**83.4 ± 9.9**

*Calculated using Cockcroft–Gault equation, based on total body weight.

^Calculated using Modification of Diet in Renal Disease equation (Levey et al., 2007).

SD, standard deviation.

#### Population Pharmacokinetic Model

A one-compartment model was preferentially selected to describe our data (justification provided as **Supplementary Method**). The parameter estimates of the final model are presented in **Table 2**, demonstrating a population clearance of 1.30 L/h [relative standard error (RSE): 7%]. The population Vd was 1.23 L/kg (RSE: 5%). CrCl significantly influenced the clearance variability of vancomycin (**Supplementary Figures S1 and S2**). All other potential covariates tested were insignificant.

**Table 2:** Population parameter estimates of the final model.

Parameter	Final estimate	RSE%	Shrinkage%	Bootstrap estimate	95% Confidence interval
Cl (L/h)	1.30	7.2	–	1.30	(1.18–1.60)
θ_CrCl_	0.023	24.2	–	0.023	(0.011–0.033)
Vd (L/kg)	1.23	4.9	–	1.23	(1.12–1.37)
ηCl (%)	54.4	10.2	12.0	53.8	(41.2–65.5)
ηVd (%)	22.5	17.1	26.0	21.9	(12.6–29.3)
Additive error (mg/L)	2.46	12.3	–	2.44	(1.85–2.98)
Proportional error (ㅘσ^2^)	0.001	–	–	–	–

RSE, Relative standard error.

**Figure 1:**
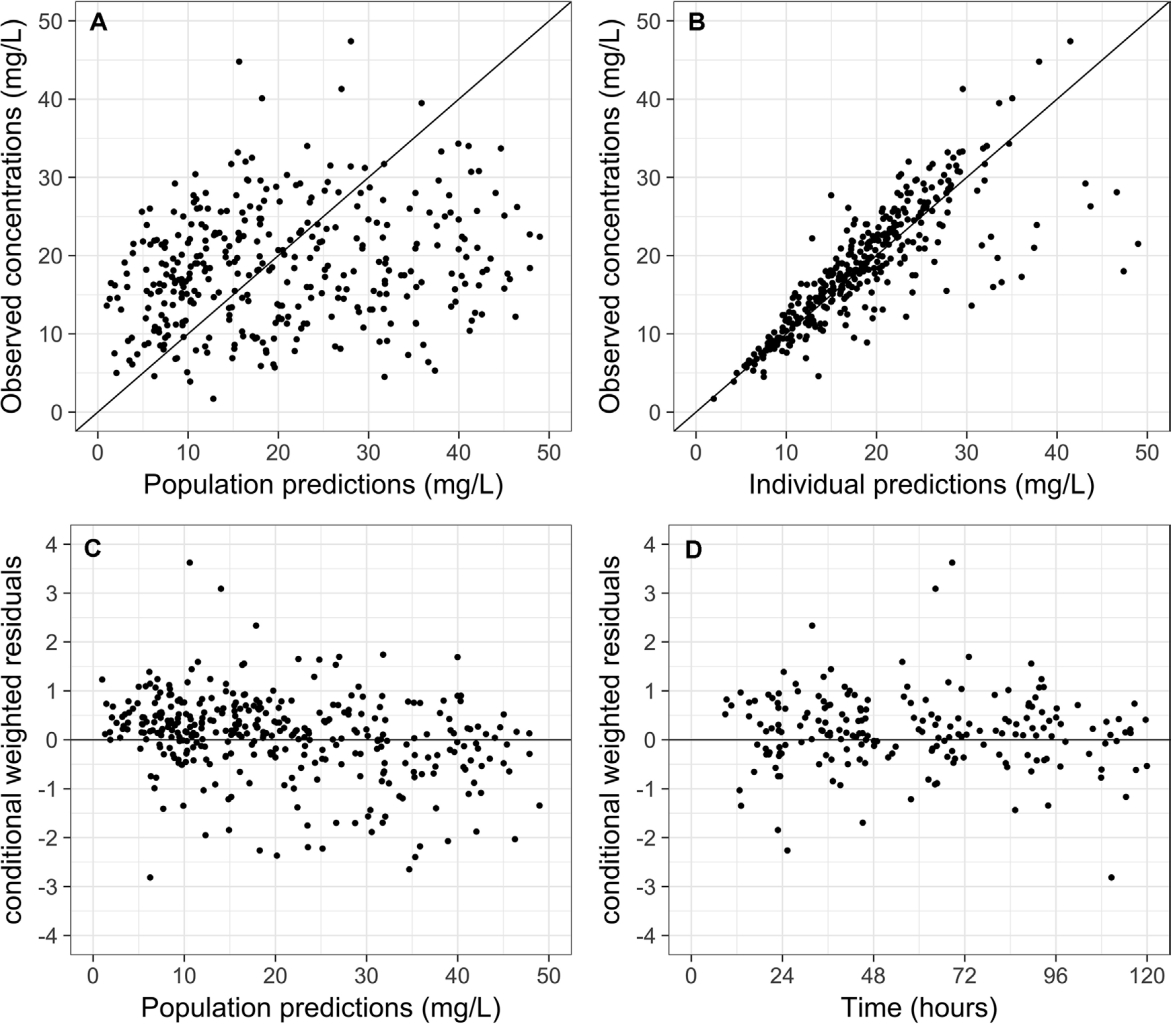
Scatter plots of **(A)** observed versus population predictions and **(B)** individual predictions of vancomycin concentrations using the final model. Scatter plots of conditional weighted residuals versus **(C)** population predictions and **(D)** time.

Scatter plots of observed versus predicted concentrations using the final model are shown in **Figure 1A and B**, respectively. The inclusion of IIV reduces the dispersion of observed versus predicted concentrations around the line of identity. Visual inspection of the CWRES versus population predictions and time after dose suggested an absence of model misspecification, with more than 95% of residuals falling within −2 and 2 (**Figure 1C and D**). The consistency estimates from bootstrapping suggested that the final model provided a satisfactory description of the data used for model construction (**Table 2**).

### Phase 2: External Validation and Web Application Development

The validation cohort consisted of 112 separate patients with a mean CrCl of 35.5 ± 13.6 ml/min and TBW of 58.7 ± 14.1 kg. As with patients in the derivation cohort, three quarters of the validation cohort comprised of patients in CKD stage 3 and beyond. Other demographic and clinical parameters were comparable (**Table 1**). A total of 289 vancomycin concentrations were acquired over the initial 120 h of therapy, amounting to an average of 2.3 ± 1.3 (range: 1–5) concentrations. The model had a MAE of 1.25 mg/L and a MSE of 1.78 (mg/L)^2^ when used to predict 289 observed concentrations in the validation cohort (other validation metrics provided in **Supplementary Table 1**).

**Figure 2:**
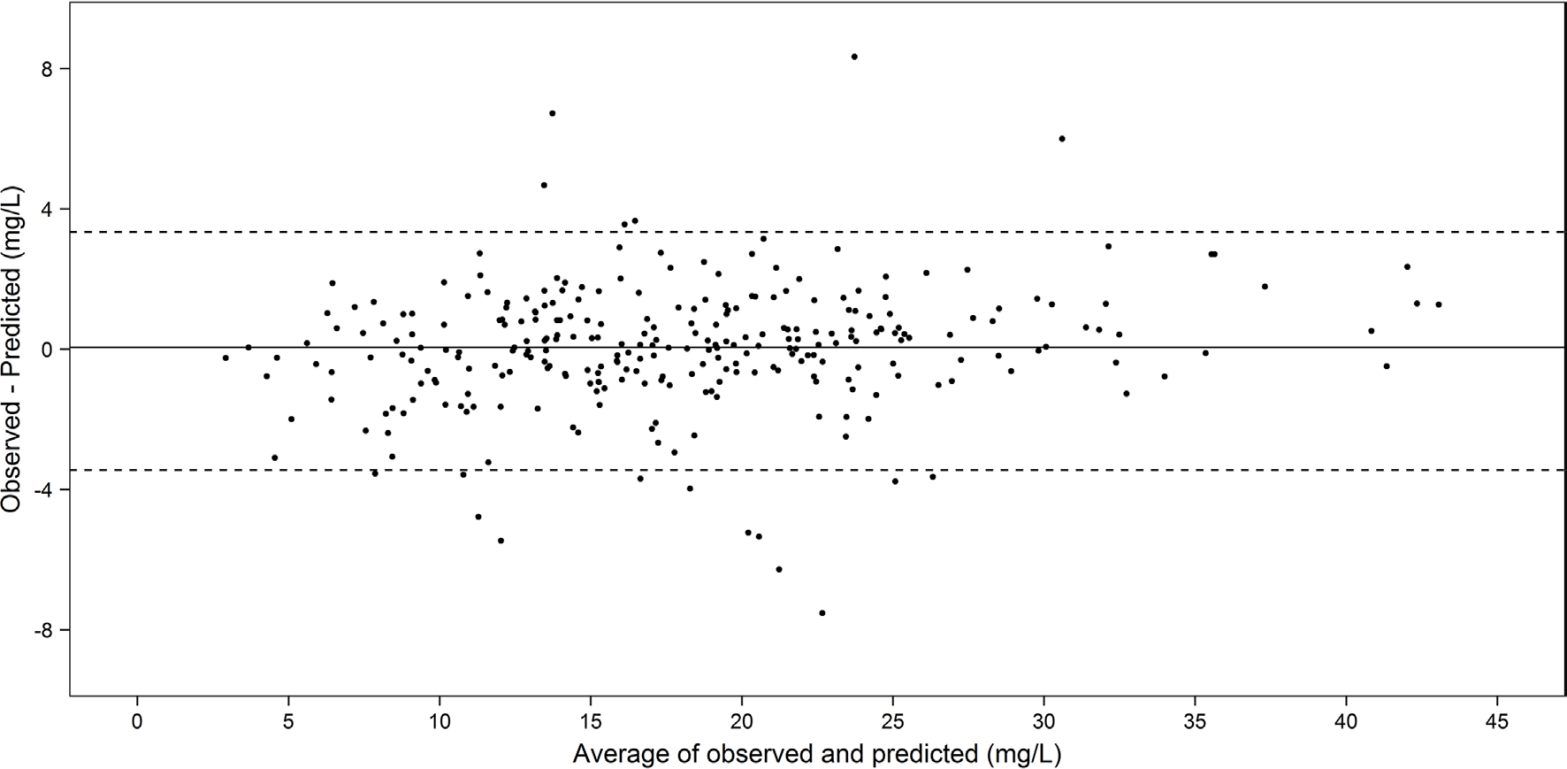
Bland–Altman plot of agreement between the observed and individual predicted concentrations in the temporal validation cohort (n = 112, 289 concentrations).

**Figure 2** is a Bland–Altman plot depicting the degree of agreement between observed and individual predicted concentrations on validation, illustrating that the vast majority of the differences between observed and predicted concentrations fall within ± 2 standard deviations (limits of agreement) of the mean difference. The differences between observed and predicted concentrations appeared consistent over the range of average concentration levels and there was no clear evidence of proportional error in the plot. Visual inspection of the Visual Predictive Check (VPC) plot is provided in **Supplementary Figure S3**. The VPC indicates that the 95% confidence band captures most of the observed concentration in the validation cohort.

Considering the results of external validation in totality, the model was deemed sufficiently accurate and precise to consider progression into the next phases of productization and clinical implementation.

The productized final models (VancApp) can be accessed at http://bit.ly/vancapp_initial and at http://bit.ly/vancapp_adjust for initial dosing and post-trough adjustments, respectively (**Supplementary Figure S4**). Initial dosing decisions were individualized and heavily depended upon patient factor such as illness severity (to determine loading dose requirement) as well as factors that could significantly affect PK parameters including creatinine clearance, total body weight, age, and gender. Care teams comprising of physicians and pharmacists had undergone briefing sessions on iteratively adjusting doses and dosing intervals that led to trough concentrations that they were intending to attain for their patient. Alternatively, clinicians could also assess their planned prior dosing regimen and evaluate the simulated concentration–time profile of vancomycin in their patient and amend it accordingly. Based on our in-house vancomycin protocol, more frequent trough monitoring is recommended in patients with renal impairment but this decision is typically left to the team’s discretion who may decide to monitor trough concentrations more/less frequently given individual patient circumstances. Following this trough measurement, dose adjustments may or may not be warranted using the adjustment module of the app, depending on the urgency to attain target concentrations.

The code to generate the initial dosing application is also provided as **Supplementary Material** (**Supplementary R code**).

### Phase 3: Clinical Implementation and Real-World Impact Assessment

A total of 256 patients received vancomycin between September 2016 and January 2017, of whom 54 were included for analysis. Twenty-two patients (41%) were dosed using VancApp from time of vancomycin initiation. Twenty-one patients (39%) were dosed according to usual care based on the in-house protocol. The remaining 11 patients (20%) had switched to VancApp-based dosing after initially receiving doses based on usual care (**Supplementary Figure S5**). The VancApp group and the usual care group had comparable gender ratios, age, total body weight, CrCl, and proportion of patients at various CKD stages**. All other **demographic parameters are compared in **Table 3**. No significant differences in terms of baseline characteristics were observed between the two groups.

**Table 3:** Characteristics of patients who had received model-based dosing and usual care-based dosing.

Variable	VancApp-based dosing (*n* = 22)	Usual care-based dosing (*n* = 21)	P-value
**Gender, n (%)**			
**Male**	**12 (55) **	**16 (76)**	**0.243**
**Female**	**10 (46)**	**5 (24)**	
**Age, years**,** mean ± SD**	**73.5 ± 11.2**	**70.4 ± 13.9**	**0.584**
**Total body weight (kg)**,** mean ± SD**	**60 ± 15.0**	**57.3 ± 12.9**	**0.576**
**Height (m)**,** mean ± SD**	**160.0 ± 8.4**	**161.7 ± 6.2**	**0.478**
**Body Mass Index (kg/m2)**,** mean ± SD**	**23.2 ± 4.3**	**21.7 ± 4.1**	**0. 274**
**Serum creatinine (µmol/L)**,** mean ± SD**	**168.4 ± 113.5**	**198.1 ± 141.1**	**0.481**
Creatinine clearance* (ml/min),** mean ± SD**	**33.5 ± 12.0**	**31.4 ± 14.5**	**0.544**
**Estimated glomerular filtration rate^ (ml/min)**,** mean ± SD**	**46.5 ± 25.6**	**46.5 ± 28.6**	**0.895**
**Intensive care unit admission, n (%)**	**3 (14)**	**4 (20) **	**0.631**
**Chronic kidney disease stage^, n (%)**			
**CKD 1**	**1 (5)**	**2 (10)**	**0.645**
**CKD 2**	**5 (23)**	**6 (29)**	
**CKD 3a**	**3 (14)**	**2 (10)**	
**CKD 3b**	**8 (36)**	**3 (14)**	
**CKD 4**	**2 (9)**	**4 (19)**	
**CKD 5**	**3 (14)**	**4 (19)**	
**Comorbidities**			
**Diabetes**	**9 (40)**	**7 (30)**	** 0.607**
**Hypertension**	**12 (55)**	**10 (50)**	**0.650**
**Malignancy**	**2 (9)**	**2 (10)**	**0.961**
**Gastrointestinal**	**3 (14)**	**2 (10)**	**0.674**
**Pulmonary**	**3 (14)**	**4 (20)**	**0.631**
**Cardiovascular**	**8 (40)**	**11 (50)**	**0.290**
**Central nervous system**	**3 (14)**	**5 (24) **	**0.391**
**Type of infection, n (%)**			
**Urinary tract infection**	**3 (14)**	**3 (14)**	**0.951**
**Community acquired pneumonia**	**2 (9)**	**6 (29)**	**0.116**
**Hospital acquired pneumonia**	**3 (14)**	**4 (19)**	**0.631**
**Soft tissue and/or skin infection**	**9 (40)**	**4 (19)**	**0.131**
**Sepsis**	**2 (9)**	**4 (19)**	**0.378**
**Bacteremia**	**1 (5)**	**3 (14)**	**0.335**
**Pyrexia of unknown origin**	**2 (9)**	**1 (5)**	**1.000**
**CNS infection**	**1 (5)**	**0 (0)**	**1.000**
**Osteomyelitis**	**2 (9)**	**0 (0)**	**0.488**
**Cholangitis**	**1 (5)**	**0 (0)**	**1.000**
**Concurrent antimicrobials**			
**β-lactams**			
**Piperacillin-tazobactam**	**10 (48)**	**12 (57)**	**0.443**
**Amoxicillin-clavulanate **	**8 (38)**	**4 (19) **	**0.310**
**Ceftazidime **	**3 (14)**	**2 (10)**	**1.000**
**Carbapenems**	**6 (29)**	**6 (29)**	**0.924**
**Floroquinolones **	**1 (5)**	**1 (5)**	**1.000**
**Aminoglycosides **	**5 (24)**	**6 (29)**	**0.660**
**Others**	**5 (24)**	**6 (29)**	**0.660**
**Concurrent nephrotoxins**			
**Vasopressors**	**3 (14)**	**3 (14)**	**1.000**
**Diuretics**	**6 (29)**	**7 (33)**	**0.665**
**ACE inhibitors/ARBs **	**5 (24)**	**6 (29)**	**0.660**
**NSAIDs **	**2 (9)**	**3 (14) **	**0.664**

^Calculated using Modified Diet Renal Disease equation.

SD, standard deviation.

#### Difference in Time-to-Target Trough Concentrations

**Figure 3:**
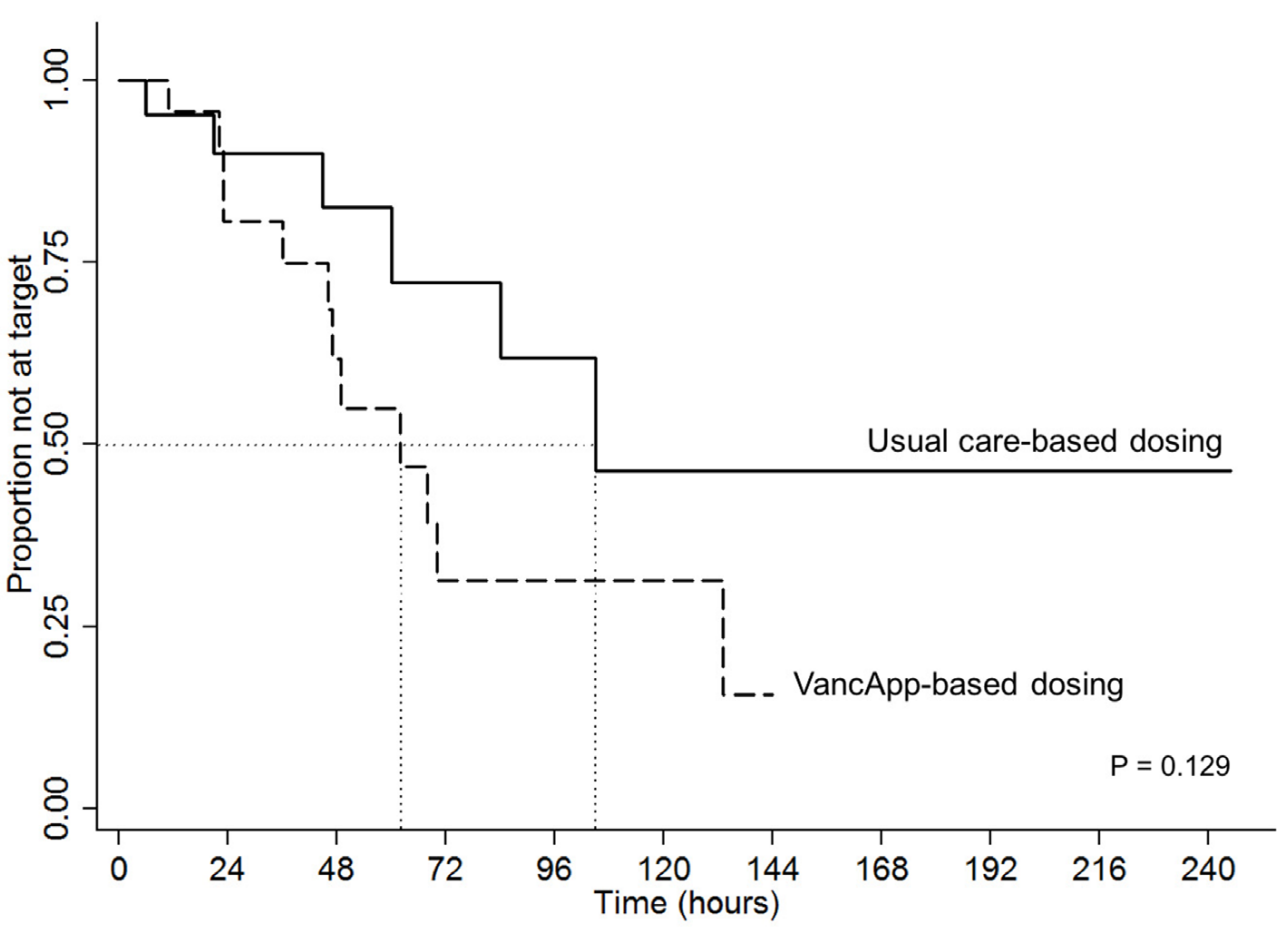
Kaplan–Meier curve comparing time to first therapeutic target between the model-based group (n = 22) and usual care group (n = 21).

**Figure 3** illustrates superimposed Kaplan–Meier curves for time taken to reach the first therapeutic vancomycin concentration within the target range of 10 to 15 or 15 to 20 mg/L. The median time to attain the first target trough was 66 h for the VancApp group versus 102 h in the usual care group. After 72 h of vancomycin therapy, 70% of the VancApp group had at least one trough within target, compared to 30% in the usual care group. These differences, however, did not attain statistical significance (log-rank test: p = 0.129).

#### Differences in Clinical Outcomes

The clinical outcomes of patients are summarized in **Table 4** by group. In light of the small sample, only unadjusted estimates are presented. The VancApp group had a higher mean (SD) length of hospitalization of 22.5 (10.7) days compared to the usual care group of 17.3 (8.4) days (p = 0.197). The VancApp group had a lower 30-day mortality (n = 3, 14%) than the usual care group (n = 5, 24%) (p = 0.457). None of the patients in the model-based group required 30-day readmission due to MRSA infection, whereas two patients (10%) dosed according to usual care were readmitted (p = 0.114). There were two patients (9%) who developed nephrotoxicity in the VancApp group versus one patient on usual care (5%). Notably, the VancApp group received a mean vancomycin dose of 22.4 ± 3.2 mg/kg/day compared to 27.7 ± 4.1 mg/kg/day in the usual care group. All three patients who developed nephrotoxicity had received concomitant piperacillin–tazobactam.

**Table 4:** Clinical outcomes of patients receiving VancApp-based dosing (n = 22) and patients dosed according to usual care (n = 21), as well as prediction accuracy of the model (n = 33, 78 concentrations).

	VancApp-based dosing	Usual care-based dosing	P-value
Clinical outcomes	(n = 22)	(n = 21)	
Nephrotoxicity, n (%)	2 (9)	1 (5)	1.000
Length of hospitalization (days), mean **± SD**	22.5 **± 10.7**	17.3 **± 8.40**	0.197
30-day mortality, n (%)	3 (14)	5 (24)	0.457
30-day readmission, n (%)	0 (0)	2 (10)	0.114
Prediction accuracy	(n = 33*)		
Mean absolute error	3.65	–	–
Mean squared error	21.33	–	–
Root mean squared error	4.62	–	–
Mean absolute prediction error (%)	32.87	–	–

*11 additional patients switched over to VancApp-based dosing mid-way through treatment period.

#### Prediction Accuracy of VancApp

On implementation, 78 vancomycin trough concentrations were obtained from the 33 patients who had received VancApp-based dosing. VancApp demonstrated a MAE of 3.65 mg/L and RMSE of 4.62 mg/L (**Table 4**) in this group.

## Discussion

CKD patients requiring intravenous vancomycin are at a pronounced risk of succumbing to adverse outcomes from both the infection as well as from inappropriate doses of vancomycin itself. Precise dosing of vancomycin in CKD is thus needed to maximize treatment success. We constructed a population pharmacokinetic model (Phase 1) for dosing vancomycin in CKD and retrospectively validated it in a separate cohort of CKD patients (Phase 2). The model was subsequently productized into a Web application (VancApp) and offered in routine practice. In Phase 3, we evaluated the impact of the VancApp-based dosing on key clinical outcomes. Our findings suggest that VancApp-based dosing can accelerate target exposures, potentially improving 30-day mortality and 30-day readmission due to MRSA infections.

The estimated population Vd of 1.30 L/kg of the model is larger than most previous estimates that have ranged between 0.4 and 1.0 L/kg, but still comparable to that identified in other PopPk analyses that had included CKD patients as part of their study population (Llopis-Salvia and Jimenez-Torres, 2006; del Mar Fernandez de Gatta Garcia et al., 2007; Rybak et al., 2009). Thomson et al. (2009) used a two-compartment model and estimated the Vd of the central and peripheral compartments to be 0.675 and 0.732 L/kg, respectively (steady state Vd = 1.41 L/kg), on a cohort consisting of patients with and without CKD having a median age of 66 years (range: 16 to 97 years) and a median total body weight of 72 kg (range: 40 to 159 kg). Although our estimated Vd is similar, our cohort consisted of older (median: 75 years, range: 31 to 97 years) and lighter****(median: 55.8 kg, range: 33.6–103.8 kg**) patients. Besides age-related changes, the higher **Vd could be attributed to chronic inflammation in CKD due to proinflammatory cytokine production, hypoalbuminemia, and acidotic conditions in addition to infection-related alterations (Tonelli et al., 2005; Verbeeck and Musuamba, 2009; Dungey et al., 2013; Akchurin and Kaskel, 2015).

Using the derived parameters and the availability of computational resources at the point-of-care, VancApp offers the ability to simulate and visualize concentration–time profiles for selected doses in individual patients. It therefore facilitates precise dose adjustments, providing suggestions to relevant questions such as how long doses should be withheld if troughs are supratherapeutic and by how much should doses be increased by if subtherapeutic.

Patients dosed with VancApp attained target concentrations approximately 36 h earlier compared to patients dosed by usual care. Timeliness of target trough attainment is an important surrogate for treatment success particularly in CKD, which prolongs the time needed to attain steady-state concentrations because of the longer half-life owing to slower clearance. Renal impairment independently raises mortality risk in severe infections such as MRSA bacteremia (Pastagia et al., 2012). It is therefore imperative to optimize doses of vancomycin in CKD patients to maximize treatment success. To this end, the model demonstrates potential for considerable clinical impact by potentially shortening the time to attain target concentrations and therefore target exposures.

Rapid attainment of initial target troughs is known to be associated with a decrease in readmission and mortality, which our findings also seem to suggest (Aliberti et al., 2011). MRSA infections have been associated with mortality rates of 20–30% (Moise-Broder et al., 2004; Soriano et al., 2008). Our findings suggest that VancApp-based dosing potentially reduces 30-day mortality, from 24% to 14%. While not statistically significant, this was observed despite the relatively older group of patients in our study population [median (interquartile range): 74 (16)], given that age is known predictor of mortality in MRSA infections (Gasch et al., 2013).

Likewise, readmission due to MRSA infections is an undesirable outcome. Besides the development of resistance and treatment failure, readmissions inevitably add to the demand for hospitalized care, unnecessarily burdening healthcare resources (Hidayat et al., 2006; Moise et al., 2007; Soriano et al., 2008). Readmissions may be indicative of incomplete bacteriological cure during the preceding admission, which could be related to dosing imprecision and delays in target attainment. The absence of 30-day readmissions in the VancApp group could be attributed to earlier target trough attainment. However, we note that there was a signal toward increased length of hospitalization in the app-based dosing arm. While the longer hospitalization may be explained partly by the lower mortality which indirectly implies that patients may be alive for a longer period, this remains to be studied more closely in a larger, prospective follow-up study.

Nonetheless, the observed improvements in clinical outcomes of 30-day readmission and 30-day mortality had occurred without a substantial rise in nephrotoxicity with vancomycin use in CKD patients, who are more vulnerable toward nephrotoxicity with vancomycin use [n = 2 (9%) in the model-based group versus n = 1 (5%) in the usual care group]. While the one additional case of nephrotoxicity in the model-based group was observed, patients who received model-based doses had received lower vancomycin doses overall (by approximately 5 mg/kg) compared to usual care. All three patients who developed nephrotoxicity were receiving concomitant piperacillin–tazobactam, which may exacerbate nephrotoxicity risks (Burgess and Drew, 2014). The nephrotoxicity rates in this study are within literature reported rates, ranging between 5% and 25% (Iwamoto et al., 2003; Hazlewood et al., 2010).

The strengths of this study include the validation assessment conducted in an independent cohort of patients and an evaluation of the productized model on important clinical outcomes following implementation. These data are lacking in the clinical pharmacokinetic literature. This study also demonstrates the value the population pharmacokinetic method. Nonetheless, this study has a number of limitations. Firstly, our initial analysis was based on retrospective data predominantly comprising of trough concentrations. Measuring peak vancomycin concentrations has been discouraged because of its poor correlation with clinical outcomes and is therefore rarely measured in our practice (Rybak et al., 2009). Although the estimated Vd appears to be reasonable for the CKD population, sparse sampling of peak concentrations in our study could have led to misspecification of the Vd as well as a preferential fit of a one-compartment model. However, the external validation and the postimplementation evaluation findings suggest that deep pharmacokinetic characterization may not be absolutely necessary to derive models that may yield clinical benefit. However, this warrants future investigation. Secondly, trough-based dose optimization can lead to higher-than-necessary vancomycin exposures which could inadvertently predispose patients toward vancomycin-related toxicities (Neely et al., 2014). Modern dose optimization methods based on 24-h area under the concentration–time curve to MIC (AUC/MIC ratio) may alter the way vancomycin is dosed in future (Pai et al., 2014). Nonetheless, inasmuch as trough-based dose optimization are still used because of their simplicity and familiarity, our findings are likely to have immediate practice implications.

Thirdly, this study did not monitor the extent and number of dose adjustments, frequency of dosage adjustments, and trough monitoring in the usual care arm to compare these criteria between the app-based dosing and usual care which have been considered and compared in previous studies (Cardile et al., 2015; Miller et al., 2018). While a more rapid attainment of target trough concentrations may indirectly indicate a reduced need for frequent adjustments and monitoring as suggested in Cardile et al. who studied an adult population, the same trends were not seen in Miller et al. who studied individualized vancomycin dosing in a pediatric population. Nonetheless, this remains to be confirmed for our app-based dosing strategy in a larger prospective validation study. Lastly, this study has been performed at a single center, comprising of a multi-ethnic Asian cohort. In general, the geographical validity of vancomycin dosing models has been dismal (Murphy et al., 2006; Deng et al., 2013). This could reflect the need for setting-specific dosing aids for vancomycin given its narrow therapeutic window and need for nuanced dose adjustments; however, a formal study of our app-based dosing in an external setting in future would need to be carried out to assess its generalizability.

Precise vancomycin dosing is important to maximize treatment success in patients with CKD. Yet CKD alters vancomycin pharmacokinetics, making precise dosing a challenge. We report the derivation, validation, and postimplementation evaluation of a vancomycin dosing application (VancApp) for optimizing dosing decisions in patients with CKD. Patients who had received VancApp-based doses had taken a shorter median time to attain target concentrations by one and a half days. While causality cannot be attributed in this nonrandomized study, corroborating trends were observed in terms of improved 30-day mortality and 30-day readmission due MRSA infections in VancApp-dosed patients.

## Data Availability Statement

Publicly available datasets were analyzed in this study. This data can be found here: https://ckdvancaid.shinyapps.io/Initial_CKD-VancAid/

## Ethics Statement

This study was carried out in three phases. The first phase involved model construction and internal validation, the second phase involved external model validation and web-application development, and the third phase involved model implementation and post-implementation evaluation. The results of each phase motivated subsequent phases of the study. All study phases were approved by the Domain Specific Review Board, with a waiver of informed consent. All phases of the study were conducted at Khoo Teck Puat Hospital (KTPH), Singapore—a 600-bed tertiary healthcare institution providing a wide range of medical and surgical specialty services.

## Author Contributions

SRD and CLW wrote the manuscript. SRD, JHFG, SYL, CWY, AC and J-WC designed the study. SRD, CLW, STO, JS, LYY performed the research. SRD, CLW, STO and J-WC analyzed the data.

## Funding

This research was supported by the National Research Foundation of Korea (NRF-2018R1C1B5085278). 

## Conflict of Interest Statement

The authors declare that the research was conducted in the absence of any commercial or financial relationships that could be construed as a potential conflict of interest.

## Supplementary Material

Table S1 Validation performance of final model on temporal validation cohort (n = 112, 289 concentrations).

Figure S1 The covariate effect of creatinine clearance on vancomycin clearance resulting in an absolute change in the typical value of vancomycin clearance. The shaded region and bars represent a 20% change in the typical value of clearance and the 95% confidence intervals of clearance change, relative to each extreme of creatinine clearance in the cohort.

Figure S2 Simulated concentration–time profiles of patients with varying degrees of estimated creatinine clearances following the administration of 1 gram of vancomycin every 24 hourly.

Figure S3 Visual predictive check of model on the external validation cohort (n = 112).

Figure S4 Screenshots of the clinical decision support Web application interfaces for initial and adjusted dosing of vancomycin in CKD.

Figure S5 Flow diagram illustrating derivation of the final cohort in impact assessment phase of the study.
