# Supplementary material for: Optimizing Vancomycin Dosing in Chronic Kidney Disease by Deriving and Implementing a Web-Based Tool Using a Population Pharmacokinetics Analysis
Source: Front Pharmacol. 2019 Jun 11;10:641. doi: 10.3389/fphar.2019.00641 (PMC6581063; doi:10.3389/fphar.2019.00641)
Supplement: Table S1 — Validation performance of final model on temporal validation cohort (n = 112, 289 concentrations). [file Table_1.docx]

**Supplementary Methods**

*List of covariates evaluated during model construction*

The following variables were collected to identify important covariates that may influence the clearance and volume of distribution (Vd) of vancomycin: age, gender, TBW, height, body mass index, body surface area, serum creatinine, CrCl (based on Cockroft-Gault and Jelliffe equations), estimated glomerular filtration rate (eGFR) based on the Modification of Diet in Renal Disease (MDRD) and the Chronic Kidney Disease Epidemiology Collaboration equations (CKD-EPI), serum albumin, C-reactive protein, procalcitonin, total white blood cell count and neutrophil percentage prior to therapy initiation

*Details of covariate selection procedure*

The ability of the covariates to explain a portion of the pharmacokinetic variability for vancomycin was evaluated using stepwise univariate forward selection followed by backward elimination. Reduction in the objective function value (OFV) was used to guide the selection of continuous and categorical covariates in the final model until no further reduction in OFV was observed. Covariates were included in the model when a significant reduction in OFV was observed (p<0.05 for 1 degree of freedom). Continuous variables were entered as linear, power and exponential functions while categorical variables were entered as linear and exponential functions. Backward elimination was performed by sequentially removing covariates from the model (p<0.01 for 1 degree of freedom) to confirm the relevance of a given covariate.[1] Significant covariates were included in the final model. In addition, the distribution of change in the typical value of a given PK parameter was derived based on extreme values of the relevant covariate distribution to assess the effect size of the covariate. An absolute change greater than 20% was considered significant.[2]

*Code for creating the initial dosing application*

The R codes for the initial dosing application were developed by adapting the code provided in the tutorial by Wojciechowski et al.[3] These are to be saved as 2 separate files (ui.R and server.R) and run on R. The exact codes are provided in the Supplementary_material_Rcode.pdf file.

**Supplementary results**

*Justification for 1-compartment model selection*

The OFVs of the one- and two-compartment models were 704.5 vs 700.6, respectively and considered similar. The Aikaike’s criterion revealed a lower value for the one-compartment model over the two-compartment model (744.5 versus 764.6). The estimates of shrinkage for inter-individual variability on clearance and Vd for 1 compartment base model were low to moderate with values of 8% and 28%, respectively. In contrast, the estimates of shrinkage for IIV on clearance and Vd for 2 compartment base model were low to high with values of 9% and 36%, respectively. In terms of precision, the relative standard error (RSE) values of 1 compartment model were generally lower than those of 2 compartment model.

**Supplementary Tables and Figures**

1Table S1. Validation performance of final model on temporal validation cohort (n=112, 289 concentrations)

| Measure | Estimate |
| --- | --- |
| Mean absolute error (mg/L) | 1.25 |
| Mean squared deviation (mg/L) | 1.62 |
| Mean squared error (mg/L)^2^ | 3.18 |
| Root mean squared error (mg/L) | 1.78 |
| Prediction error range (%) | (-39.3 – 103.1) |
| Mean absolute prediction error (%) | 8.6 |

**
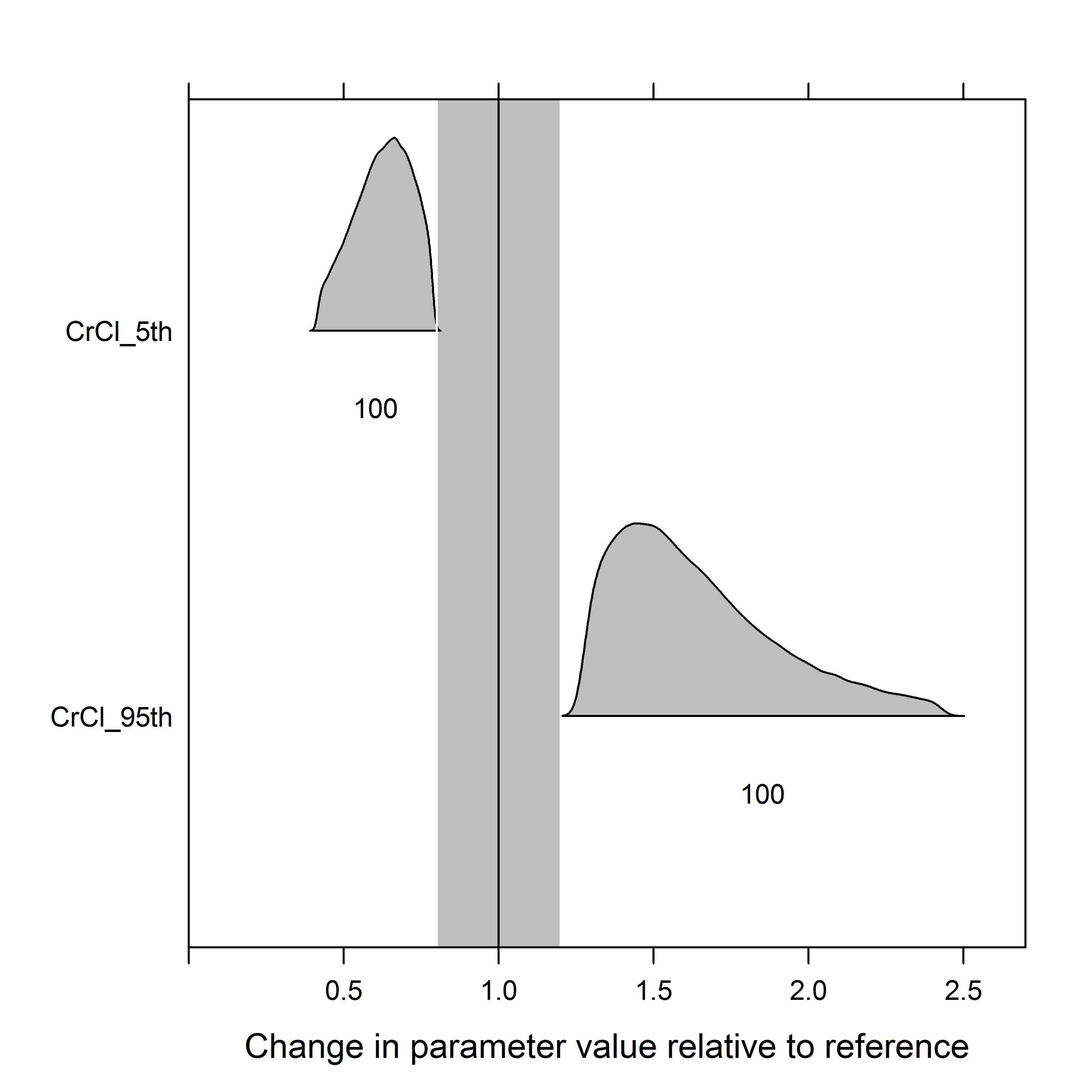
**

2Figure S1. The covariate effect of creatinine clearance on vancomycin clearance resulting in an absolute change in the typical value of vancomycin clearance. The shaded region and bars represent a 20% change in the typical value of clearance and the 95% confidence intervals of clearance change, relative to each extreme of creatinine clearance in the cohort.

**
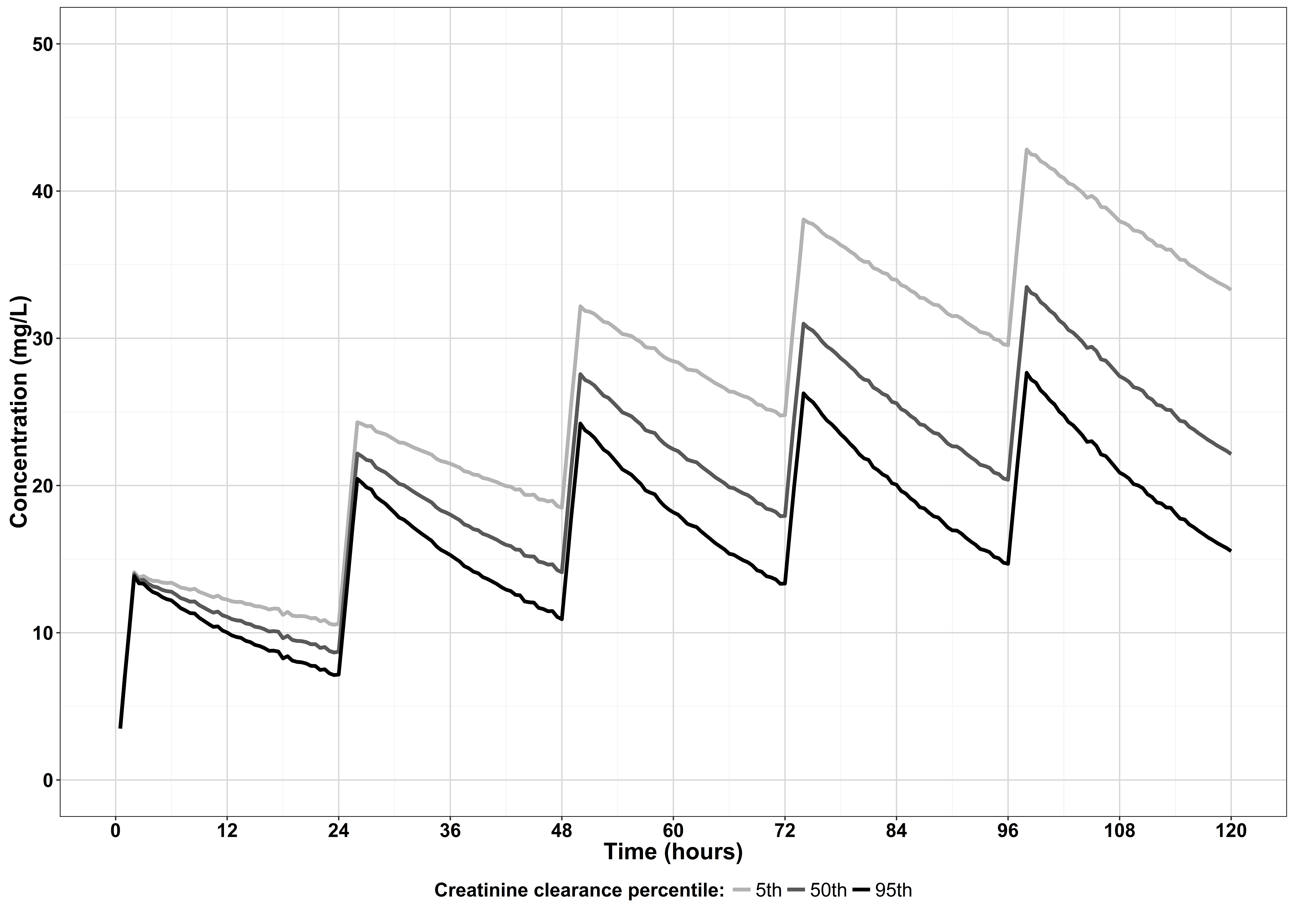
**

Figure S2. Simulated concentration-time profiles of patients with varying degrees of estimated creatinine clearances following the administration of 1 gram of vancomycin every 24 hourly.


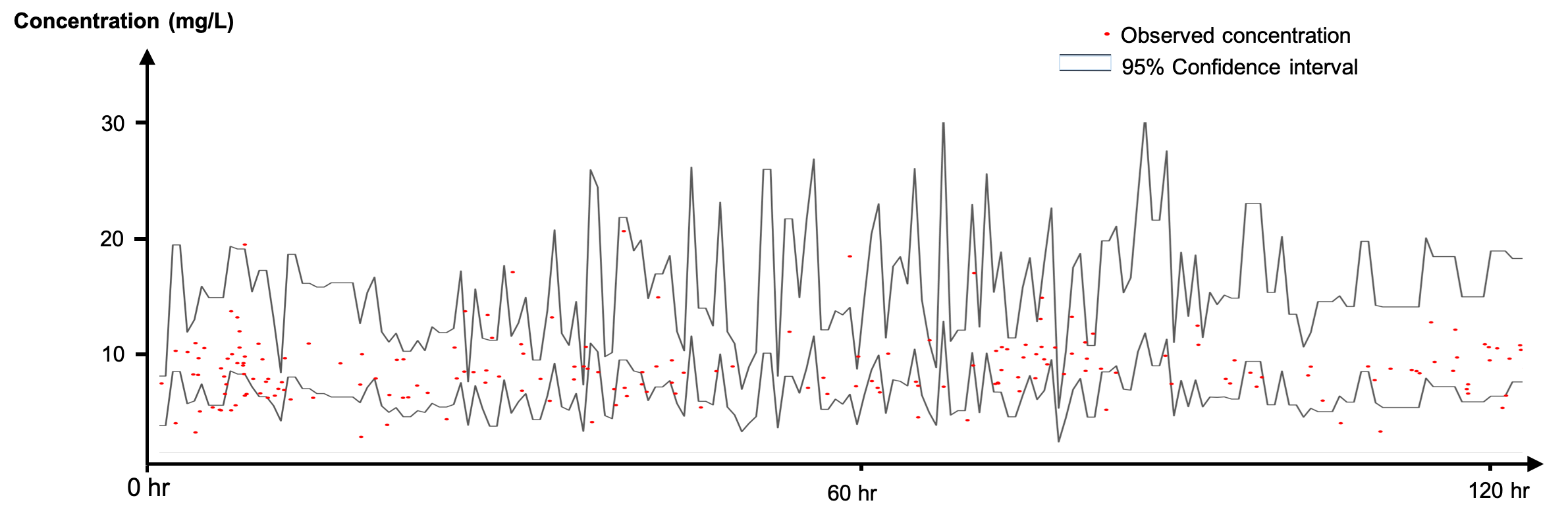


Figure S3. Visual predictive check of model on the external validation cohort (n=112).

**
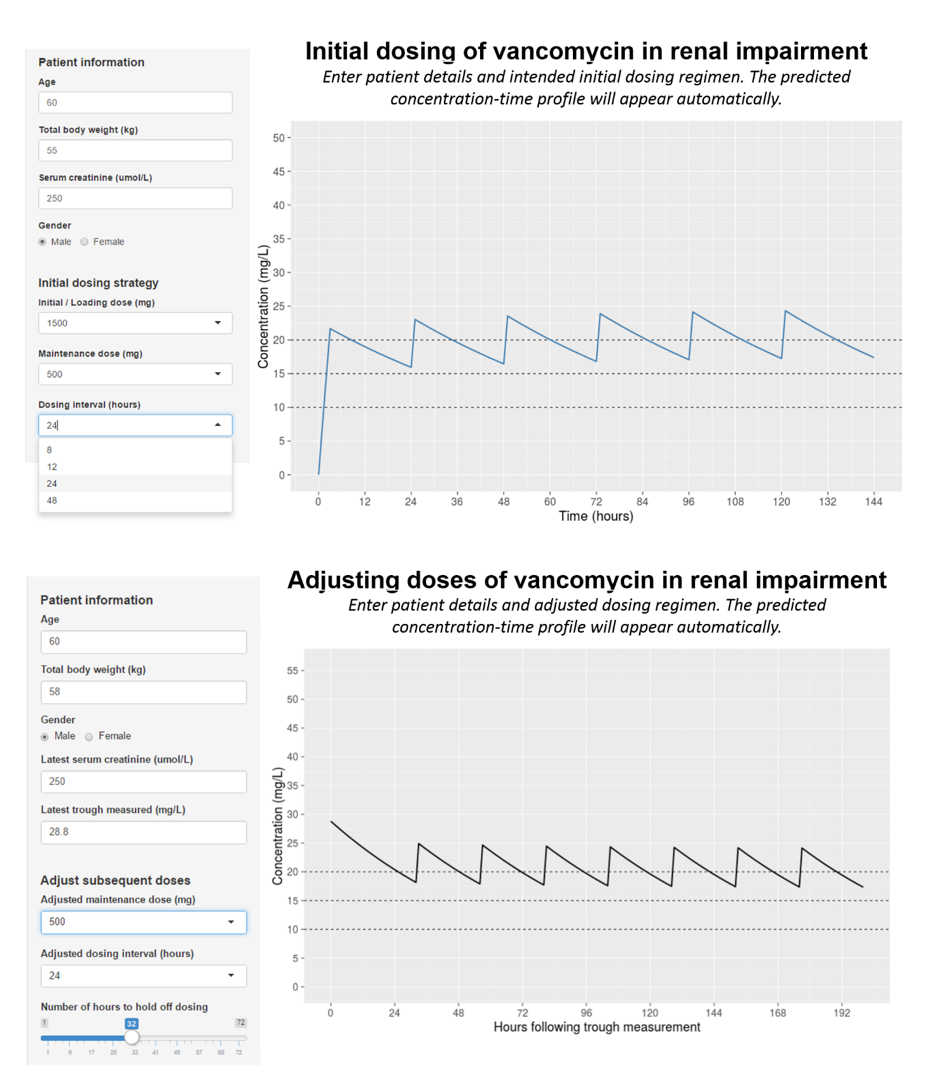
**

Figure S4. Screenshots of the clinical decision support web application interfaces for initial and adjusted dosing of vancomycin in CKD.


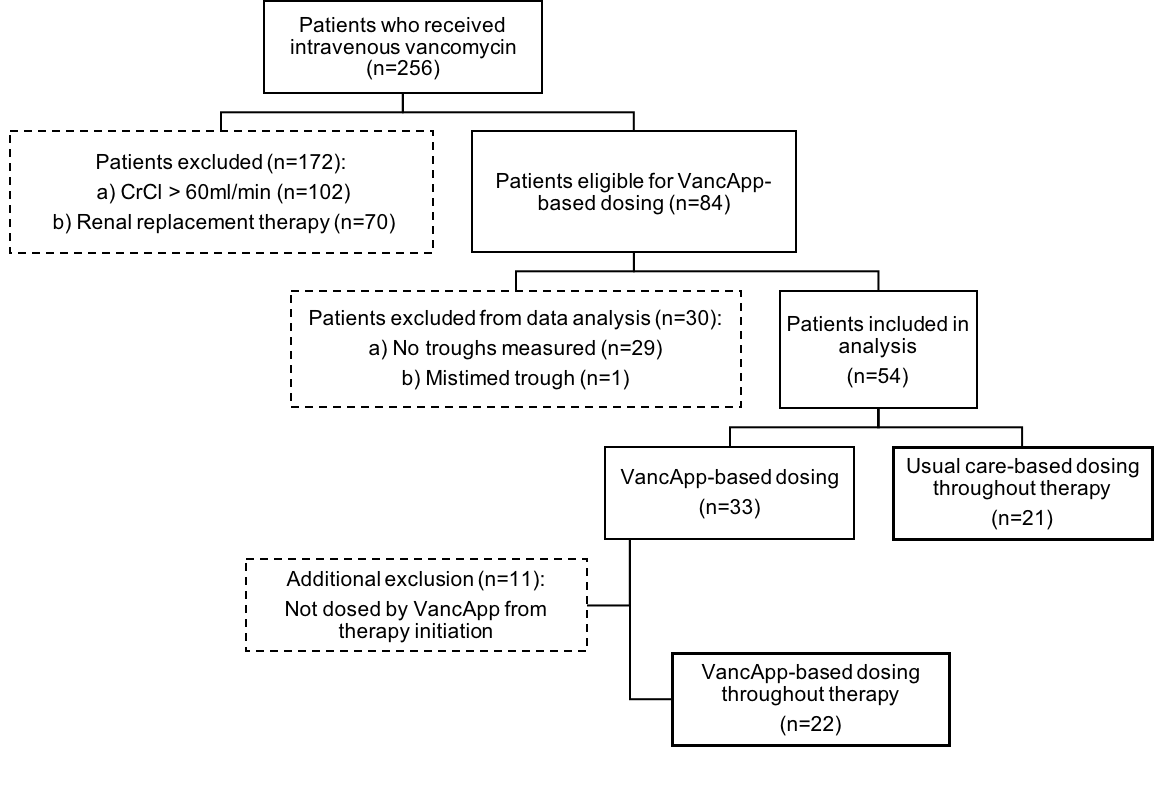


2Figure S5. Flow diagram illustrating derivation of the final cohort in impact assessment phase of the study.

**References**

1. Yano, Y., S.L. Beal, and L.B. Sheiner, *Evaluating pharmacokinetic/pharmacodynamic models using the posterior predictive check.* J Pharmacokinet Pharmacodyn, 2001. **28**(2): p. 171-92.

2. Moore, J.N., et al., *A Population Pharmacokinetic Model for Vancomycin in Adult Patients Receiving Extracorporeal Membrane Oxygenation Therapy.* CPT Pharmacometrics Syst Pharmacol, 2016. **5**(9): p. 495-502.

3. Wojciechowski, J., A.M. Hopkins, and R.N. Upton, *Interactive Pharmacometric Applications Using R and the Shiny Package.* CPT Pharmacometrics Syst Pharmacol, 2015. **4**(3): p. e00021.
