# Supplementary material for: Optimizing Vancomycin Dosing in Chronic Kidney Disease by Deriving and Implementing a Web-Based Tool Using a Population Pharmacokinetics Analysis
Source: Front Pharmacol. 2019 Jun 11;10:641. doi: 10.3389/fphar.2019.00641 (PMC6581063; doi:10.3389/fphar.2019.00641)
Supplement: Supplementary file 2 [file DataSheet_1.pdf]

```
## Supplementary R code for Initial dosing application

# UI.R

#INITIAL DOSING APPLICATION

fluidPage(

  #Application Title and Logo
  fixedRow(
    column(9,
          titlePanel("Initial dosing of vancomycin in renal impairment*"),
          h5("Dorajoo SR, Leandra CW, Goh HFJ, Ooi ST, Somani J, Lee SY, Yap CY, Chan A and
Chae JW,"),
          div("*Patients with CrCl < 60 mL/min NOT receiving renal replacement therapy",style
= "color:red"),
          offset = 2, align = "center")
  ),       #Brackets closing "fixedRow"

  hr(),   #Add a break with a horizontal line
  #Sidebar panel with widgets
  sidebarLayout(
    sidebarPanel(width = 3,

               #Selection box for dosing regimen

               #PATIENT SPECIFIC INFORMATION
               h4(strong("Patient information")),


               textInput("AGE", "Age", width = 300,"60"),

               textInput("WT","Total body weight (kg)", width = 300,"65"),

               textInput("SCR", "Serum creatinine (umol/L)", width = 300,"250"),

               radioButtons("SEX", "Gender",list("Male","Female"),"Male", inline=TRUE),
               br(),

               #DOSING SPECIFIC INFORMATION
               h4(strong("Initial dosing strategy")),

               selectInput(inputId = "LDOSE", label = "Initial / Loading dose (mg)", width = 300,
choices = c("None" = 0 , "250" = 250 , "500" = 500 , "750" = 750, "1000" = 1000 , "1250" =
1250, "1500" = 1500 , "1750" = 1750, "2000" = 2000), selected = 1500),
```

```
              selectInput(inputId = "MDOSE", label = "Maintenance dose (mg)",  width =
300,choices = c("0" =0 , "250" = 250 ,"500" = 500 , "750" = 750 , "1000" = 1000 , "1250" =
1250, "1500" = 1500, "1750" = 1750, "2000" = 2000 ),selected = 500),

              selectInput(inputId = "FREQ", label = "Dosing interval (hours)",  width =
300,choices = c("8"= 8 ,"12" = 12, "24" = 24, "48" = 48), selected = 24),


              #Selection box for prediction intervals



              #Button to initiate simulation
              #submitButton("Simulate"),

              align = "left"),          #Brackets closing "sidebarPanel"

    mainPanel(
      h4(strong("Patient information"), align = "left"),

      textOutput("AGE"), align = "left",
      textOutput("SEX"), align = "left",
      textOutput("WT"), align = "left",
      textOutput("SCR"), align = "left",

      textOutput("CRCL"), align = "left",
      textOutput("t_half"),
      br(),
      div(textOutput("WARNING"), style = "color:red"),

      #div("div creates segments of text with a similar style. This division of text is all blue
because I passed the argument 'style = color:blue' to div", style = "color:blue"),

      h4(strong("Initial dosing strategy")),

      textOutput("LDOSE"),
      div(textOutput("LdoseWARNING"), style = "color:red"),
      textOutput("MDOSE"),
      div(textOutput("MdoseWARNING"), style = "color:red"),
      textOutput("FREQ"),


      #Plot output for concentration-time profile
      plotOutput("plotCONC", height = 630, width = 1000


      ),  align = "center"
```

```
    )       #Brackets closing "mainPanel"
  )         #Brackets closing "sidebarLayout"
)           #Brackets closing "fluidPage"
```

```
# SERVER.R

#INITIAL DOSING APPLICATION

#Load package libraries
library(shiny)
library(ggplot2)
library(deSolve)
library(plyr)
library(reshape2)
library(compiler)
library(foreach)

#ggplot2 theme for plotting
theme_custom <- theme_set(theme_grey(20))

#TIME range - times where a concentration will be calculated
TIME <- seq(from = 0, to = 144, by = 0.5)

#-----------------------------------------------------------------------------------------------------------
#Define user-input dependent functions for output
shinyServer(function(input, output) {

  #Reactive expression to generate a reactive data frame
  #This is called whenever the input changes
  all.data <- reactive({

#Function for calculating median, upper and lower confidence intervals for x

    sumfuncx <- function(x) {
      stat1 <-  median(x)
      stat2 <- median(x) +2.5
      stat4 <-  length(x)
      result <- c("median"=stat1,"median25"= stat2, "n"=stat4)  # optional low and hi for error
bars
      result
    }


    SEX <- as.character(input$SEX)
    AGE <- as.numeric(input$AGE)
    WT <- as.numeric(input$WT)
    SCR <- as.numeric(input$SCR)
```

```
#------------------------------------------------------------------------------------------
#Create a dataframe with ID and parameter values for each individual
#Number of individuals
# n <- input$n [fixed at 100 for reaction speed and reasonable error bars]
n=100
par.data <- seq(from = 1, to = n, by = 1)
par.data <- data.frame(par.data)
names(par.data)[1] <- "ID"

#Make a parameter vector for input into DES function
#par.data$K21 <- 0.014      #hour^-1

#Define population values
POPV1 <- 0.95        #mL/kg
POPCL <- 1.4
# POPKE1 <- 0.0253           #hour^-1


#Define population parameter variability
ETA1 <- rnorm(n, mean = 0, sd = 0.0855)
ETA3 <- rnorm(n, mean = 0, sd = 0.202)


#Simulate individual values
par.data$V1 <- POPV1*WT*exp(ETA1)               #mL/kg
par.data$CL <- POPCL*exp(ETA3)
# par.data$CL <- POPCL*exp(ETA3)          #hour^-1


#------------------------------------------------------------------------------------------

#Input dosing data for loading dose (LDOSE = loading dose)
LDOSE <- as.numeric(input$LDOSE)                 #microg/kg
LTinf <- LDOSE/500                               #Loading dose infusion duration (hours)
LRATE <- LDOSE/LTinf                       #Loading infusion rate

LTIMEinf <- c(0,LTinf,150)             #Vector marking infusion's time events, function
#works long after infusion is finished
LRATEinf <- c(LRATE,0,0)               #Vector marking infusion's rates

#Define an interpolation function that returns rate when given time - "const"
Lstep.doseinf <- approxfun(LTIMEinf, LRATEinf, method = "const")

# Frequency of maintenance doses -
FREQ <- as.numeric(input$FREQ)
```

```
   #Input dosing data for 2 bolus doses (BDOSE = bolus dose)
   MDOSE <- as.numeric(input$MDOSE)                  #microg/kg
   MTinf <- MDOSE/500                                        #Bolus dose infusion duration
(hours)
   MRATE <- MDOSE/MTinf                          #Bolus infusion rate


   if (FREQ==48) {
     MTIMEinf <- c(0,       48,  MTinf+48,   96, MTinf+96,   144, MTinf+144,    150)     #Vector
marking infusion's time events, function

     MRATEinf <- c(0,    MRATE,        0,           MRATE,        0, MRATE, 0,0 )             #Vector
marking infusion's rates
   }

   if (FREQ==24) {
     MTIMEinf <- c(0,       24,  MTinf+24,              48,  MTinf+48,       72, MTinf+72,    96,
MTinf+96,   120, MTinf+120, 144, MTinf+144,           150)     #Vector marking infusion's time
events, function

     MRATEinf <- c(0,    MRATE,        0,           MRATE,        0,                    MRATE,       0,
MRATE,       0, MRATE,        0, MRATE, 0,  0)            #Vector marking infusion's rates
   }

   if (FREQ==12) {
     MTIMEinf <- c(0, 12,MTinf+12, 24,MTinf+24, 36,MTinf+36, 48,MTinf+48, 60,MTinf+60,
72,MTinf+72, 84,MTinf+84, 96,MTinf+96, 108,MTinf+108, 120,MTinf+120,132,MTinf+132,
144,MTinf+144,           150)     #Vector marking infusion's time events, function

     MRATEinf <- c(0, MRATE,0, MRATE,0, MRATE,0, MRATE,0,  MRATE,0,  MRATE,0,
MRATE,0, MRATE,0, MRATE,0, MRATE,0,MRATE,0,MRATE,0,  0)                #Vector marking
infusion's rates
   }

   if (FREQ==8) {
     MTIMEinf <- c(0, 8,MTinf+8, 16,MTinf+16, 24,MTinf+24, 32,MTinf+32, 40,MTinf+40,
48,MTinf+48, 56,MTinf+56, 64,MTinf+64, 72,MTinf+72, 80,MTinf+80, 88,MTinf+88,
96,MTinf+96, 104,MTinf+104, 112,MTinf+112, 120,MTinf+120, 128, MTinf+128,
136,MTinf+136, 144, MTinf+144,       150)     #Vector marking infusion's time events,
function

     MRATEinf <- c(0, MRATE,0, MRATE,0, MRATE,0, MRATE,0,  MRATE,0,  MRATE,0,
MRATE,0, MRATE,0, MRATE,0, MRATE,0, MRATE,0, MRATE,0, MRATE,0, MRATE,0, MRATE,0,
MRATE,0,MRATE,0,MRATE,0,0)                    #Vector marking infusion's rates
   }
```

```
   #Define an interpolation function that returns rate when given time - "const"
   Mstep.doseinf <- approxfun(MTIMEinf, MRATEinf, method = "const")

   #Input patient data
   SEX <- as.character(input$SEX)
   AGE <- as.numeric(input$AGE)
   WT <- as.numeric(input$WT)
   SCR <- as.numeric(input$SCR)


   #CRCL
   NUM <- (140-AGE)*WT*88.4
   DENOM <- 72*SCR
   if (SEX=="Female") CRCL <- (NUM/DENOM)*0.85
   else CRCL <- (NUM/DENOM)

   V  <- 0.95*WT
   CL <- 1.4*( 1 + 0.0224*(CRCL - 35.75))
   #T1/2
   t_half <- (log(2)*V)/ CL

   output$SEX <- renderText({paste("Gender: ", input$SEX)})
   output$AGE <- renderText({paste("Age: ", input$AGE, "years")})
   output$SCR <- renderText({paste("Serum creatinine: ", input$SCR, "umol/L")})
   output$WT <- renderText({paste("Total body weight: ", input$WT, "kg")})
   output$CRCL <- renderText({paste("Creatinine clearance: ", signif(CRCL, digits = 3),
"mL/min")})

   output$t_half <- renderText({paste("Estimated half-life of vancomycin: ", signif(t_half,
digits = 3), "hours")})
   EXCEEDLDOSE <- LDOSE/WT
   EXCEEDMDOSE <- MDOSE/WT

   if (EXCEEDLDOSE > 30.0) {
     output$LdoseWARNING <- renderText(paste("*Initial / Loading dose exceeds
30mg/kg."))
   }
   else {
     output$LdoseWARNING <- renderText(paste(""))
   }

   if (EXCEEDMDOSE > 30.0) {
     output$MdoseWARNING <- renderText(paste("*Maintenance dose exceeds 30mg/kg."))
   }
   else {
     output$MdoseWARNING <- renderText(paste(""))
   }
```

```
  if (CRCL > 60.0) {
    output$WARNING <- renderText(paste("This model is has been developed for patients with a creatinine clearance lesser than 60mL/min ONLY. Caution is advised in interpreting the predicted concentration-time profile. "))
  }
  else {
    output$WARNING <- renderText(paste(""))
  }

  output$TT <- renderText({paste("Target trough range: ", min(input$tTrough), " - ", max(input$tTrough), "mg/L")})
  output$LDOSE <- renderText({paste("Initial / Loading dose: ", input$LDOSE,"mg", "(dosing at", signif(as.numeric(input$LDOSE)/WT, digits = 3) ,"mg/kg)")})
  output$MDOSE <- renderText({paste("Maintenance dose: ", input$MDOSE,"mg every", input$FREQ, "hourly", "(dosing at", signif(as.numeric(input$MDOSE)/WT, digits = 3) ,"mg/kg)")})


  #Differential equations
  DES <- function(T, A, THETA) {

    #define the rate functions
    RateL <- Lstep.doseinf(T)
    RateM <- Mstep.doseinf(T)

    KE1 <- THETA[1]
    dA <- vector(length = 1)
    dA[1] = RateL + RateM  - (CL/V)*A[1]


    list(dA)
  }
  #Compile DES function
  DES.cmpf <- cmpfun(DES)

  #------------------------------------------------------------------------------------
  #Function for simulating concentrations for the ith patient
  simulate.conc <- function(par.data) {

    #Set initial conditions in each compartment
    A_0 <- c(A1 = 0)

    #List of parameter values
    THETAlist <- c("CL"=par.data$CL)

    #Run differential equation solver for simulated parameter data
```

```
  sim.data <- lsoda(A_0, TIME, DES.cmpf, THETAlist)
  sim.data <- as.data.frame(sim.data)
 }

 #Compile simulate.conc function
 simulate.conc.cmpf <- cmpfun(simulate.conc)

 #Apply simulate.conc.cmpf function to each individual in par.data
 #ID is provided as a variable in which each value has simulate.conc.cmpf applied to
 #V1 and V2 are required to be here to preserve their values, so concentrations can be
 #calculated later
 sim.data <- ddply(par.data, .(ID,V1), simulate.conc.cmpf, .parallel = TRUE)

 #Calculate concentration
 sim.data$CONCS <- sim.data$A1/sim.data$V1


 statsCONCS <- ddply(sim.data, .(time), function(sim.data) sumfuncx(sim.data$CONCS))
 names(statsCONCS)[c(2,3)] <- c("Smedian","Smedian25")



 for (i in 1:nrow(statsCONCS)) {
  if (i > 1) {
   statsCONCS$Smedian1[i] = statsCONCS$Smedian[i] + 2.5
  }

  else {
   statsCONCS$Smedian1[i] = statsCONCS$Smedian[i]
  }

 }

 #Combine both datasets
 sim.data <- statsCONCS

})      #Brackets closing "reactive" expression

#------------------------------------------------------------------------------------
#Generate a plot of the data
#Also uses the inputs to build the plot


output$plotCONC <- renderPlot({

 plotobj <- ggplot(all.data())
```

```
    plotobj <- plotobj + geom_abline(aes(slope = 0, intercept = 10), linetype = "dashed", size =
0.5)
    plotobj <- plotobj + geom_abline(aes(slope = 0, intercept = 15), linetype = "dashed", size =
0.5)
    plotobj <- plotobj + geom_abline(aes(slope = 0, intercept = 20), linetype = "dashed", size =
0.5)

    plotobj <- plotobj + geom_line(aes(x = time, y = Smedian1), colour = "#4682b4", size = 1)
    #plotobj <- plotobj + geom_line(aes(x = time, y = Smedian1), colour = "#4682b4", size = 1)



    plotobj <- plotobj + scale_y_continuous("Concentration (mg/L)", lim=c(0,50), breaks=
seq(from = 0, to = 50, by = 5))
    plotobj <- plotobj + scale_x_continuous("Time (hours)", breaks=seq(from = 0, to = 144, by
= 12))

    print(plotobj)

  })        #Brackets closing "renderPlot" function

})        #Brackets closing "shinyServer" function
```
